# (*E*,*E*)-1,2-Bis(2,4,5-trimeth­oxy­benzyl­idene)hydrazine

**DOI:** 10.1107/S1600536811019040

**Published:** 2011-05-25

**Authors:** Hoong-Kun Fun, Patcharaporn Jansrisewangwong, Chatchanok Karalai, Suchada Chantrapromma

**Affiliations:** a X-ray Crystallography Unit, School of Physics, Universiti Sains Malaysia, 11800 USM, Penang, Malaysia; b Department of Chemistry and Center of Excellence for Innovation in Chemistry, Faculty of Science, Prince of Songkla University, Hat-Yai, Songkhla 90112, Thailand; c Crystal Materials Research Unit, Department of Chemistry, Faculty of Science, Prince of Songkla University, Hat-Yai, Songkhla 90112, Thailand

## Abstract

The asymmetric unit of the title compound, C_20_H_24_N_2_O_6_, contains one half-mol­ecule, the complete mol­ecule being generated by a crystallographic inversion centre. The mol­ecule is nearly planar with a dihedral angle between the two benzene rings of 0.03 (4)° and the central C/N/N/C plane making a dihedral angle of 8.59 (7)° with each of its two adjacent benzene rings. The two meth­oxy groups at the *ortho* and *meta* positions are slightly twisted [C—O—C—C torsion angles = 7.23 (12) and 5.73 (13)°], whereas the meth­oxy group at the *para* position is almost coplanar with the attached benzene ring [C—O—C—C torsion angle = −2.02 (13)°]. The crystal structure is stabilized by a weak C—H⋯π inter­action.

## Related literature

For bond-length data, see: Allen *et al.* (1987 ▶). For related structures, see: Fun *et al.* (2010 ▶); Jansrisewangwong *et al.* (2010 ▶); Zhao *et al.* (2006 ▶). For background to and the bio­logical activity of hydrazones, see: Avaji *et al.* (2009 ▶); El-Tabl *et al.* (2008) ▶; Kitaev *et al.* (1970 ▶); Qin *et al.* (2009 ▶); Ramamohan *et al.* (1995 ▶); Rollas & Küçükgüzel (2007 ▶). For the stability of the temperature controller used in the data collection, see: Cosier & Glazer (1986 ▶).
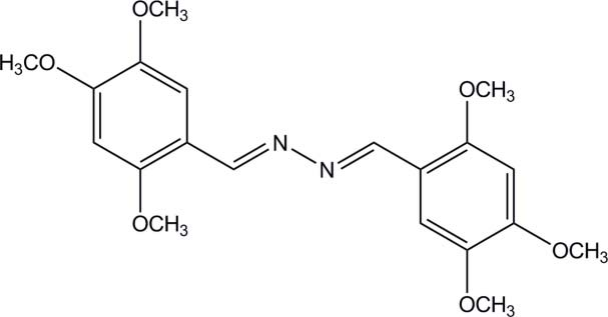


## Experimental

### 

#### Crystal data

C_20_H_24_N_2_O_6_
*M* *_r_* = 388.41
Monoclinic, 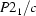

*a* = 7.5056 (1) Å
*b* = 7.2523 (1) Å
*c* = 17.4489 (2) Å
β = 90.600 (1)°
*V* = 949.74 (2) Å^3^
*Z* = 2
Mo *K*α radiation
μ = 0.10 mm^−1^
*T* = 100 K
0.47 × 0.29 × 0.10 mm

#### Data collection

Bruker APEXII CCD area-detector diffractometer
Absorption correction: multi-scan (*SADABS*; Bruker, 2005 ▶) *T*_min_ = 0.954, *T*_max_ = 0.990
18099 measured reflections
2778 independent reflections
2384 reflections with *I* > 2σ(*I*)
*R*_int_ = 0.028

#### Refinement

*R*[*F* ^2^ > 2σ(*F* ^2^)] = 0.038
*wR*(*F* ^2^) = 0.113
*S* = 1.04
2778 reflections
175 parameters
All H-atom parameters refined
Δρ_max_ = 0.45 e Å^−3^
Δρ_min_ = −0.22 e Å^−3^

### 

Data collection: *APEX2* (Bruker, 2005 ▶); cell refinement: *SAINT* (Bruker, 2005 ▶); data reduction: *SAINT*; program(s) used to solve structure: *SHELXTL* (Sheldrick, 2008 ▶); program(s) used to refine structure: *SHELXTL*; molecular graphics: *SHELXTL*; software used to prepare material for publication: *SHELXTL* and *PLATON* (Spek, 2009 ▶).

## Supplementary Material

Crystal structure: contains datablocks global, I. DOI: 10.1107/S1600536811019040/is2713sup1.cif

Structure factors: contains datablocks I. DOI: 10.1107/S1600536811019040/is2713Isup2.hkl

Supplementary material file. DOI: 10.1107/S1600536811019040/is2713Isup3.cml

Additional supplementary materials:  crystallographic information; 3D view; checkCIF report

## Figures and Tables

**Table 1:** Hydrogen-bond geometry (Å, °) *Cg*1 is the centroid of the C1–C6 ring.

*D*—H⋯*A*	*D*—H	H⋯*A*	*D*⋯*A*	*D*—H⋯*A*
C8—H8*C*⋯*Cg*1^i^	0.974 (13)	2.675 (14)	3.4837 (10)	140.7 (10)

Symmetry code: (i) 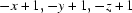.

**Fig. 1.**
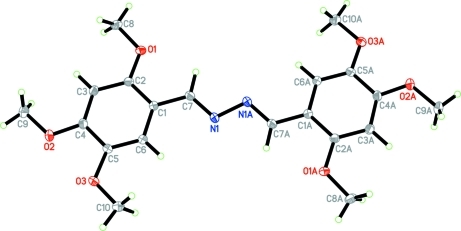
The molecular structure of the title compound, showing 50% probability displacement ellipsoids and the atom-numbering scheme. Atoms with suffix A were generated by symmetry code -x, -y, 1 - z.

**Fig. 2.**
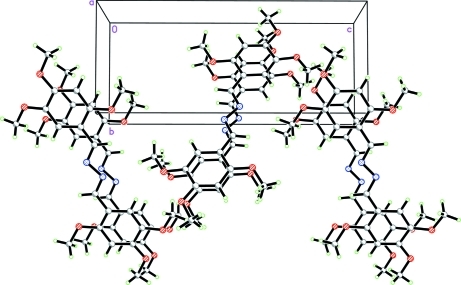
The crystal packing of the title compound viewed along the a axis, showing screw chains running along the c axis and stacked along the a axis.

## supplementary crystallographic information

### Comment

Hydrazones, which is a class of compounds containing the >C=N—N=C< (Avaji *et al.*, 2009), have been studied for their fluorescence properties (Qin *et al.*, 2009) and biological activities such as insecticides, antitumor agents, antioxidants (Kitaev *et al.*, 1970), antimicrobial (Ramamohan *et al.*, 1995) and antiviral properties (El-Tabl *et al.*, 2008; Rollas & Küçükgüzel, 2007). We have previously reported the crystal structure of (*E*,*E*)-1,2-bis(2,4,6-trimethoxybenzylidene)hydrazine (Fun *et al.*, 2010). In this work, 2,4,5-trimethoxy substituents on the benzene ring was used in order to get information about the effect of trimethoxy substituent positions on the fluorescence property of the compound. Herein we report the synthesis and crystal structure of the title compound (I).

The asymmetric unit of (I) (Fig. 1), C_20_H_24_N_2_O_6_, contains one half-molecule and the complete molecule is generated by a crystallographic inversion centre -*x*, -*y*, 1 - *z*. The molecule of (I) exists in an *E*,*E* configuration with respect to the two C═N double bonds [1.2870 (12) Å] and the torsion angle N1A–N1–C7–C1 = -178.99 (9)°. The molecule is nearly planar with the dihedral angle between the two benzene rings being 0.03 (4)°. Atoms C7/N1/N1A/C7A lie on a same plane [*r.m.s* 0.000 (1) Å]. This C/N/N/C plane makes a dihedral angle of 8.59 (7)° with each of its two adjacent benzene rings. The three methoxy groups of the 2,4,5-trimethoxyphenyl unit have two different orientations: two methoxy groups at the *ortho* and *meta* positions (at atom C2 and C5 positions) are slightly twisted with the attached benzene ring with torsion angles C8–O1–C2–C3 = 7.23 (12)° and C10–O3–C5–C6 = 5.73 (13)° whereas the third one at *para* position (at atom C4) is co-planarly attached with the torsion angle C9–O2–C4–C3 = -2.02 (13)°. The bond distances are of normal values (Allen *et al.*, 1987) and are comparable with related structures (Fun *et al.*, 2010; Jansrisewangwong *et al.*, 2010; Zhao *et al.*, 2006).

In the crystal structure (Fig. 2), the molecules are arranged into screw chains along the *c* axis and these chains stacked along the *a* direction. the molecules are consolidated by C—H···π (Table 1) and π–π interactions with the *Cg*1···*Cg*1 distances of 4.6314 (5) Å (symmetry code: -*x*, 1 - *y*, 1 - *z*) and 4.9695 (5) Å (symmetry code: 1 - *x*, 1 - *y*, 1 - *z*). C···C [3.3411 (12)–3.3987 (12) Å] short contacts were observed.

### Experimental

The title compound was synthesized by mixing a solution (1:2 molar ratio) of hydrazine hydrate (0.097 ml, 2 mmol) and 2,4,5-trimethoxybenzaldehyde (0.785 mg, 4 mmol) in ethanol (20 ml). The resulting solution was refluxed for 5 h, yielding the yellow solid. The resultant solid was filtered off and washed with methanol. Yellow block-shaped single crystals of the title compound suitable for *x*-ray structure determination were recrystallized from acetone by slow evaporation of the solvent at room temperature over several days, m.p. 523 K (decompose).

### Refinement

All H atoms are located from a difference map and refined isotropically [refined distances: C—H = 0.924 (13)–0.995 (12) Å].

### Crystal data

**Table:**

C_20_H_24_N_2_O_6_	*F*(000) = 412
*M**_r_* = 388.41	*D*_x_ = 1.358 Mg m^−^^3^
Monoclinic, *P*2_1_/*c*	Melting point = 523 decompose–523 K
Hall symbol: -P 2ybc	Mo *K*α radiation, λ = 0.71073 Å
*a* = 7.5056 (1) Å	Cell parameters from 2778 reflections
*b* = 7.2523 (1) Å	θ = 2.3–30.0°
*c* = 17.4489 (2) Å	µ = 0.10 mm^−^^1^
β = 90.600 (1)°	*T* = 100 K
*V* = 949.74 (2) Å^3^	Needle, colorless
*Z* = 2	0.47 × 0.29 × 0.10 mm

### Data collection

**Table:**

Bruker APEXII CCD area-detector diffractometer	2778 independent reflections
Radiation source: sealed tube	2384 reflections with *I* > 2σ(*I*)
graphite	*R*_int_ = 0.028
φ and ω scans	θ_max_ = 30.0°, θ_min_ = 2.3°
Absorption correction: multi-scan (*SADABS*; Bruker, 2005)	*h* = −10→10
*T*_min_ = 0.954, *T*_max_ = 0.990	*k* = −10→10
18099 measured reflections	*l* = −24→24

### Refinement

**Table:**

Refinement on *F*^2^	Primary atom site location: structure-invariant direct methods
Least-squares matrix: full	Secondary atom site location: difference Fourier map
*R*[*F*^2^ > 2σ(*F*^2^)] = 0.038	Hydrogen site location: inferred from neighbouring sites
*wR*(*F*^2^) = 0.113	All H-atom parameters refined
*S* = 1.03	*w* = 1/[σ^2^(*F*_o_^2^) + (0.0652*P*)^2^ + 0.2144*P*] where *P* = (*F*_o_^2^ + 2*F*_c_^2^)/3
2778 reflections	(Δ/σ)_max_ = 0.001
175 parameters	Δρ_max_ = 0.45 e Å^−^^3^
0 restraints	Δρ_min_ = −0.22 e Å^−^^3^

### Special details

**Table:**

Experimental. The crystal was placed in the cold stream of an Oxford Cryosystems Cobra open-flow nitrogen cryostat (Cosier & Glazer, 1986) operating at 100.0 (1) K.
Geometry. All e.s.d.'s (except the e.s.d. in the dihedral angle between two l.s. planes) are estimated using the full covariance matrix. The cell e.s.d.'s are taken into account individually in the estimation of e.s.d.'s in distances, angles and torsion angles; correlations between e.s.d.'s in cell parameters are only used when they are defined by crystal symmetry. An approximate (isotropic) treatment of cell e.s.d.'s is used for estimating e.s.d.'s involving l.s. planes.
Refinement. Refinement of *F*^2^ against ALL reflections. The weighted *R*-factor *wR* and goodness of fit *S* are based on *F*^2^, conventional *R*-factors *R* are based on *F*, with *F* set to zero for negative *F*^2^. The threshold expression of *F*^2^ > σ(*F*^2^) is used only for calculating *R*-factors(gt) *etc*. and is not relevant to the choice of reflections for refinement. *R*-factors based on *F*^2^ are statistically about twice as large as those based on *F*, and *R*- factors based on ALL data will be even larger.

### Fractional atomic coordinates and isotropic or equivalent isotropic displacement parameters (Å^2^)

**Table:**

	*x*	*y*	*z*	*U*_iso_*/*U*_eq_	
O1	0.26607 (9)	0.49751 (9)	0.57223 (4)	0.01763 (16)	
O2	0.36459 (9)	0.78767 (10)	0.32476 (4)	0.02050 (17)	
O3	0.19145 (9)	0.52720 (10)	0.25671 (4)	0.01952 (17)	
N1	0.03148 (10)	0.06200 (11)	0.47211 (4)	0.01607 (17)	
C1	0.16786 (11)	0.35906 (12)	0.45749 (5)	0.01377 (18)	
C2	0.25348 (11)	0.50724 (13)	0.49415 (5)	0.01428 (18)	
C3	0.32155 (11)	0.65346 (13)	0.45116 (5)	0.01573 (18)	
H3	0.3849 (17)	0.7563 (18)	0.4767 (7)	0.017 (3)*	
C4	0.30093 (11)	0.65370 (13)	0.37186 (5)	0.01537 (18)	
C5	0.21005 (12)	0.50854 (13)	0.33441 (5)	0.01493 (18)	
C6	0.14716 (11)	0.36283 (12)	0.37708 (5)	0.01463 (18)	
H6	0.0847 (18)	0.261 (2)	0.3529 (8)	0.027 (3)*	
C7	0.09679 (11)	0.20862 (13)	0.50302 (5)	0.01512 (18)	
H7	0.0952 (17)	0.2209 (18)	0.5580 (8)	0.024 (3)*	
C8	0.33469 (13)	0.65505 (14)	0.61202 (6)	0.0198 (2)	
H8A	0.3252 (17)	0.6258 (19)	0.6634 (8)	0.024 (3)*	
H8B	0.2642 (17)	0.767 (2)	0.5997 (8)	0.026 (3)*	
H8C	0.4583 (18)	0.6749 (19)	0.5978 (7)	0.024 (3)*	
C9	0.45348 (15)	0.94046 (15)	0.35983 (6)	0.0249 (2)	
H9A	0.3707 (19)	1.004 (2)	0.3925 (8)	0.034 (4)*	
H9B	0.5579 (18)	0.897 (2)	0.3891 (8)	0.031 (3)*	
H9C	0.4852 (18)	1.010 (2)	0.3165 (8)	0.028 (3)*	
C10	0.08467 (16)	0.39153 (16)	0.21898 (6)	0.0263 (2)	
H10A	−0.0319 (16)	0.3831 (18)	0.2454 (7)	0.019 (3)*	
H10B	0.1449 (19)	0.274 (2)	0.2202 (8)	0.032 (4)*	
H10C	0.0681 (19)	0.439 (2)	0.1671 (9)	0.036 (4)*	

### Atomic displacement parameters (Å^2^)

**Table:**

	*U*^11^	*U*^22^	*U*^33^	*U*^12^	*U*^13^	*U*^23^
O1	0.0217 (3)	0.0191 (3)	0.0121 (3)	−0.0045 (3)	−0.0015 (2)	−0.0018 (2)
O2	0.0267 (3)	0.0158 (3)	0.0191 (3)	−0.0071 (3)	0.0024 (3)	0.0024 (3)
O3	0.0275 (3)	0.0192 (4)	0.0119 (3)	−0.0045 (3)	−0.0002 (2)	0.0015 (2)
N1	0.0200 (3)	0.0150 (4)	0.0132 (3)	−0.0027 (3)	0.0013 (3)	0.0023 (3)
C1	0.0144 (4)	0.0136 (4)	0.0133 (4)	−0.0009 (3)	0.0004 (3)	0.0001 (3)
C2	0.0145 (3)	0.0155 (4)	0.0128 (4)	−0.0004 (3)	0.0001 (3)	−0.0010 (3)
C3	0.0159 (4)	0.0136 (4)	0.0178 (4)	−0.0020 (3)	0.0005 (3)	−0.0014 (3)
C4	0.0162 (4)	0.0129 (4)	0.0171 (4)	−0.0003 (3)	0.0024 (3)	0.0013 (3)
C5	0.0172 (4)	0.0156 (4)	0.0121 (4)	−0.0005 (3)	0.0010 (3)	0.0004 (3)
C6	0.0166 (4)	0.0135 (4)	0.0138 (4)	−0.0018 (3)	0.0005 (3)	−0.0004 (3)
C7	0.0168 (4)	0.0164 (4)	0.0121 (4)	−0.0013 (3)	0.0001 (3)	0.0011 (3)
C8	0.0215 (4)	0.0204 (5)	0.0173 (4)	−0.0020 (4)	−0.0017 (3)	−0.0057 (3)
C9	0.0301 (5)	0.0182 (5)	0.0266 (5)	−0.0096 (4)	0.0038 (4)	−0.0007 (4)
C10	0.0402 (6)	0.0241 (5)	0.0145 (4)	−0.0086 (5)	−0.0043 (4)	−0.0010 (4)

### Geometric parameters (Å, °)

**Table:**

O1—C2	1.3664 (10)	C4—C5	1.4112 (12)
O1—C8	1.4301 (11)	C5—C6	1.3789 (12)
O2—C4	1.3624 (11)	C6—H6	0.970 (14)
O2—C9	1.4279 (12)	C7—H7	0.964 (13)
O3—C5	1.3681 (10)	C8—H8A	0.924 (13)
O3—C10	1.4257 (12)	C8—H8B	0.991 (14)
N1—C7	1.2870 (12)	C8—H8C	0.974 (13)
N1—N1^i^	1.4103 (15)	C9—H9A	0.966 (15)
C1—C2	1.4031 (12)	C9—H9B	0.981 (14)
C1—C6	1.4102 (12)	C9—H9C	0.944 (14)
C1—C7	1.4544 (12)	C10—H10A	0.995 (12)
C2—C3	1.3987 (12)	C10—H10B	0.967 (16)
C3—C4	1.3906 (12)	C10—H10C	0.976 (15)
C3—H3	0.988 (13)		
			
C2—O1—C8	117.62 (7)	N1—C7—C1	122.08 (8)
C4—O2—C9	117.39 (7)	N1—C7—H7	119.0 (8)
C5—O3—C10	116.12 (7)	C1—C7—H7	118.9 (8)
C7—N1—N1^i^	111.49 (9)	O1—C8—H8A	104.9 (8)
C2—C1—C6	118.95 (8)	O1—C8—H8B	111.1 (8)
C2—C1—C7	119.62 (8)	H8A—C8—H8B	110.6 (11)
C6—C1—C7	121.39 (8)	O1—C8—H8C	109.5 (8)
O1—C2—C3	123.51 (8)	H8A—C8—H8C	111.4 (11)
O1—C2—C1	116.21 (8)	H8B—C8—H8C	109.4 (11)
C3—C2—C1	120.28 (8)	O2—C9—H9A	108.9 (9)
C4—C3—C2	119.81 (8)	O2—C9—H9B	110.1 (9)
C4—C3—H3	119.7 (7)	H9A—C9—H9B	111.1 (12)
C2—C3—H3	120.5 (7)	O2—C9—H9C	101.2 (9)
O2—C4—C3	124.41 (8)	H9A—C9—H9C	112.6 (12)
O2—C4—C5	115.05 (8)	H9B—C9—H9C	112.4 (12)
C3—C4—C5	120.54 (8)	O3—C10—H10A	108.7 (7)
O3—C5—C6	125.38 (8)	O3—C10—H10B	109.8 (9)
O3—C5—C4	115.40 (8)	H10A—C10—H10B	110.4 (12)
C6—C5—C4	119.22 (8)	O3—C10—H10C	104.5 (9)
C5—C6—C1	121.15 (8)	H10A—C10—H10C	110.3 (11)
C5—C6—H6	121.1 (8)	H10B—C10—H10C	112.8 (12)
C1—C6—H6	117.8 (8)		
			
C8—O1—C2—C3	7.23 (12)	C10—O3—C5—C4	−173.90 (8)
C8—O1—C2—C1	−173.26 (8)	O2—C4—C5—O3	−2.95 (12)
C6—C1—C2—O1	178.64 (7)	C3—C4—C5—O3	177.39 (8)
C7—C1—C2—O1	0.86 (12)	O2—C4—C5—C6	177.39 (8)
C6—C1—C2—C3	−1.84 (13)	C3—C4—C5—C6	−2.26 (13)
C7—C1—C2—C3	−179.61 (8)	O3—C5—C6—C1	−177.86 (8)
O1—C2—C3—C4	−179.16 (8)	C4—C5—C6—C1	1.76 (13)
C1—C2—C3—C4	1.34 (13)	C2—C1—C6—C5	0.27 (13)
C9—O2—C4—C3	−2.02 (13)	C7—C1—C6—C5	178.00 (8)
C9—O2—C4—C5	178.34 (8)	N1^i^—N1—C7—C1	−178.99 (9)
C2—C3—C4—O2	−178.90 (8)	C2—C1—C7—N1	−173.65 (8)
C2—C3—C4—C5	0.72 (13)	C6—C1—C7—N1	8.63 (13)
C10—O3—C5—C6	5.73 (13)		

Symmetry codes: (i) −*x*, −*y*, −*z*+1.

### Hydrogen-bond geometry (Å, °)

**Table:**

*Cg*1 is the centroid of the C1–C6 ring.

**Table:**

*D*—H···*A*	*D*—H	H···*A*	*D*···*A*	*D*—H···*A*
C8—H8C···Cg1^ii^	0.974 (13)	2.675 (14)	3.4837 (10)	140.7 (10)

Symmetry codes: (ii) −*x*+1, −*y*+1, −*z*+1.
